# Sociocultural barriers to hepatitis B health literacy in an immigrant population: a focus group study in Korean Americans

**DOI:** 10.1186/s12889-021-10441-4

**Published:** 2021-02-25

**Authors:** Sarah Hyun, Okhyun Ko, Soonsik Kim, William R. Ventura

**Affiliations:** 1 Columbia University Mailman School of Public Health, 722 W 168th St, New York, NY 10964 USA; 2 KCS Public Health and Research Center, 2 W. 32nd St. Suite 604, New York, NY 10001 USA; 3 St. Joseph’s Regional Medical Center, Paterson, NJ 07503 USA

**Keywords:** Chronic hepatitis B (CHB), Community health, Health disparities, Health literacy, Immigrant population

## Abstract

**Background:**

Chronic hepatitis B (CHB) is a major global health issue disproportionately affecting Asian Americans and other immigrant populations in the United States. Despite the high risk of morbid complications from CHB, the majority of individuals with CHB do not access healthcare due to a complex of barriers. These barriers influence health literacy which may affect behaviors linked to hepatitis B care. We aimed to identify and evaluate various sociocultural factors and how they interact with health literacy to impact CHB care and health seeking in a Korean American population.

**Methods:**

A total of 28 Korean American individuals with CHB were divided into 5 different focus discussion groups. This study investigated the participants’ sociocultural backgrounds as well as their awareness and utilization of the healthcare system that could influence their health literacy and behaviors in accessing care.

**Results:**

Our analysis identified and concentrated on three themes that emerged from these discussions: low risk perception and knowledge of CHB and its complications; language, immigrant status, and stigma; and financial and institutional barriers. The participants’ overall awareness of the disease and prevention methods demonstrated poor understanding of important characteristics and potential outcomes of the disease. Additionally, differences in cultural expectations and a lack of understanding and utilization of healthcare systems affected health literacy in further limiting participants’ motivation to seek care.

**Conclusions:**

The present study suggests that there are culture-specific barriers to health literacy governing individuals’ health behavior in accessing hepatitis B care. These findings may inform strategies for developing culturally tailored resources and programs and for facilitating the implementation of community-wide hepatitis B education and screening initiatives in immigrant communities.

**Supplementary Information:**

The online version contains supplementary material available at 10.1186/s12889-021-10441-4.

## Background

Chronic hepatitis B (CHB) is one of the most common infectious diseases of the liver currently affecting over 250 million people worldwide [1] and causing approximately 887,000 deaths annually from CHB complications such as liver cancer and cirrhosis [2]. CHB is also a disease of ethnic disparity as there is a marked difference in CHB prevalence and levels of access to care among various regions of the world [3–5]. For instance, the prevalence of hepatitis B virus (HBV) infection is highest in sub-Saharan Africa and East Asia, where up to 8% of the population is chronically infected [6]. By contrast, at 0.3%, HBV prevalence is lowest in Western Europe and North America [7].

HBV prevalence also varies considerably among different ethnicities within the United States (U.S.), disproportionately affecting immigrants from HBV-endemic countries. Given that approximately 50,000 people legally enter the U.S. every year from intermediate to highly endemic (i.e. HBV prevalence > 2%) countries and a majority of these individuals are not screened, CHB remains a serious public health issue in the U.S. [8, 9].

Asian Americans are highly susceptible to poor health outcomes from chronic HBV infection for the following reasons. First, of 2.2 million people chronically infected with HBV in the U.S., approximately half are Asian Americans and Pacific Islanders [10]. Surprisingly, 67% of people living with the virus do not know that they have it [11], largely due to absence of symptoms, as well as the lack of universal screening. Second, CHB is undertreated, indicated by a significant discrepancy between the number of patients receiving treatment and the number of treatment-eligible patients [12, 13]. Third, a majority of Asian Americans with CHB are infected through mother-to-child transmission, resulting in a lifetime exposure to the virus [14]. As the age at HBV infection is correlated with the prognosis of the disease, those infected perinatally have consistently shown a high probability of remaining chronically infected [15]. Fourth, Asian Americans are the most likely of any ethnic group to develop liver cancer. The reasons for this liver cancer predisposition are multifactorial. In addition to a significantly higher prevalence in CHB among Asian Americans as compared to other ethnicities, Asian Americans are exposed to the virus for a longer duration of their life course as the majority of Asian Americans are infected at a young age [16]. There are also differences in genotypes and prevalence of HBV mutations among ethnic groups that may contribute to varying degrees of carcinogenicity [17]. Despite all these risks, screening and preventative behavior in Asian Americans have been found to be infrequent and poor, respectively, resulting in low levels of healthcare utilization for consistent monitoring and treatment of CHB in the Asian American population [18].

The above observations likely relate to specific cultural and social determinants of health [19–21]. Social determinants of health are conditions in the sociopolitical environments in which people are born and live, work, and play that affect a wide range of health and quality of life outcomes. Conditions in these various settings such as availability of resources, availability of healthy foods, public safety, quality of education, and social support networks can have significant influence on health outcomes. Health is also a cultural concept, as culture shapes how individuals perceive their experiences. Cultural determinants of health encompass people’s culturally based beliefs, practices and values that impact their health outcomes. Social and cultural factors inevitably interact with biological factors to impact health, through determining a person’s experience and their definition of health [22, 23].

These sociocultural factors can also impact health literacy- a crucial element in determination of health seeking behaviors and health outcomes. Health literacy is a multidimensional skillset that significantly facilitates health management, navigation of healthcare systems and resources, and health knowledge. For the purposes of this study in probing into individual accounts, we have chosen to define health literacy as the degree to which an individual has the capacity to obtain, communicate, process and understand basic health information and services to make appropriate health decisions [24].

Health literacy improves peoples’ control over their health by identifying factors important to health outcomes. Low levels of health literacy, as demonstrated by studies in numerous diseases, affect roughly one-third of the U.S. adult population [25]. Studies found that a low level of health literacy is associated with decreased likelihood of preventative health measures [26], higher frequency of hospital admissions with increased morbidity and mortality [27], and greater risk of long-term, life-limiting health conditions, and more difficulty managing medications [28].

Poor health literacy also disproportionately affects racial and ethnic minorities, contributing to health disparities in these populations [29]. Since CHB is a disease prevalent among ethnic minorities and immigrants, many of whom do not consider English their first language and are unfamiliar with the culture and social systems of the U.S., a lack of health literacy may further worsen CHB health outcomes. Thus, a health literacy framework may be useful in identifying and evaluating the role of sociocultural barriers to care, particularly for a disease of ethnic disparity like CHB. A number of studies have shown health knowledge gaps as a critical barrier that hinders access to care in CHB [30–32]. Some other studies have also evaluated the effects of misconceptions and lower education level on healthcare access among CHB patients [33, 34]. In addition, there are limited studies accounting for health literacy and its influence on infectious disease, which have suggested an association between health literacy and protective behaviors such as immunization and antibiotic use for the treatment of infections [35].

While previous works have examined health knowledge, they have not investigated health literacy as a driving force of health behaviors in responding to CHB. The dearth of published studies on these topics highlights the need to further investigate how CHB can be better understood through sociocultural contexts within an immigrant population.

The aim of the present study was to identify and evaluate various sociocultural factors and how these interact with health literacy to impact CHB care and health seeking within a Korean American immigrant population. We carried out focus group discussions with 28 Korean American adults living with CHB. Since CHB burden in the Korean American population is heavy with a significant disparity in health access, we investigated sociocultural barriers to hepatitis B literacy and their influence on access to care.

## Methods

### Participants

Participants were Korean American adults who were found to be hepatitis B surface antigen (HBsAg) seropositive (i.e., HBV-infected) from community-based hepatitis B awareness campaigns conducted in Flushing, New York, between January 2016 and June 2017. The term “Korean Americans” refers to those who were either born in Korea or in the United States with parents from Korea. These community-based campaigns consisted of CHB education and screening programs led by Korean Community Services (KCS). KCS is a non-profit organization with a broad range of programs that address various needs of the community. These include programs devoted to promoting CHB screening and linkage to care in the Korean American community. We collaborated with KCS to further investigate barriers to health literacy and CHB care as they had access to a group of Korean Americans with CHB who were already participants in their events.

A total of 52 people with CHB were identified and invited to participate in this study. They were given a written description of the study, which included the study’s purpose and procedure. Twenty-eight of 52 signed up for the study. The inclusion criteria required that participants be Korean American adults and hepatitis B surface antigen positive. The exclusion criteria were if the participants had any condition that would limit their ability to participate in the study or if they refused to give informed consent. Before the study, participants signed an informed consent form available in both Korean and English. No incentives were provided for the participants to join the study.

### Study design

Twenty-eight participants were randomly divided into 5 groups, each consisting of 5 or 6 individuals. The focus groups provided opportunities for the moderators to probe into specific topics and related matters on which the participants showed interest in elaborating on. Prior to each session, moderators briefly described the contents of discussion materials to participants. Each of the focus group discussions took 2 to 2 ½ hours. Focus group sessions took place at a conference room within the KCS headquarters located in Flushing, New York. Eight participants were fluent in both Korean and English, and all participants preferred to communicate in Korean.

Focus groups were identified as the best approach to investigate the topic of interest for a number of reasons. As focus groups are ideal for gaining multiple perspectives on a shared experience, they helped gauge a wide variety of experiences with CHB, as all participants were living with CHB. Focus groups also helped elucidate commonalities and differences between participants’ experiences. The focus group questions were less oriented toward individual-specific information but more toward the range of experiences, opinions, and concerns of the group. In this group environment, the moderator and participants shifted from being passive observers to active listeners, gaining the chance to reflect on and assess the issues being raised. The focus groups facilitated a multi-faceted exploration of CHB-related inquiries. Specifically, the participants were asked about barriers to CHB care, their experiences with healthcare providers, and their understanding of transmission risks and potential outcomes of CHB.

In this study, focus groups revealed a fluid dynamic in which participants exchanged and tested out ideas with each other, spurring conversation on new topics. There was very little potential for sensitive health information to be revealed based on the fact that none of the questions on the question guide prompted participants to reveal sensitive personal information. It was also emphasized that the participants did not have to share anything that would make them feel uncomfortable.

Participants were ensured anonymity going into the study, and all collected data were anonymized prior to analysis. There was a possibility that the participants could have known each other in the community prior to the focus group, but this was not the case for this study.

### Data collection and analysis

All participants filled out a survey assessing demographic and epidemiologic characteristics [see Additional file 1]. The items in the survey included gender, date of birth, country of birth, contact information and preferred language. The questionnaire and screening procedures were reviewed and approved by an Institutional Review Board. All data were anonymized before review and analysis. All sessions were audio-recorded and transcribed by investigators.

A pilot codebook was generated based on themes that the researchers identified in and derived from the transcriptions. The investigators then independently applied the codebook to the transcripts in order to modify and reconcile the final version with appropriate codes and sub-codes. It was inductive content analysis as the investigators worked from individual and group accounts in the focus group to develop themes. Inconsistencies between coders were examined and debated within the team of investigators and resolved unanimously. Discrepancies were discussed thoroughly as each investigator explained their rationale for coding and the group voted to decide which code to use. The group was able to come to a clear resolution for all discrepancies.

## Results

### Demographic and epidemiologic characteristics of participants

The sample of 28 individuals in the present cohort included 16 men and 12 women whose ages ranged from 20 to 69, with a mean age of 54 (Table 1). All subjects were Korean Americans currently living in the state of New York. Twenty-two (79%) of 28 were born in Korea and 6 in the U.S. A majority of the participants, 24 (86%) of 28, had been living in the U.S. for over 10 years.

**Table 1:** Demographics of HBsAg-seropositive participants

	Number of participants	Percentage (%)
Gender		
Male	16	57
Female	12	43
Age Groups		
20–39	8	29
40–49	10	36
50–59	6	21
60–69	4	14
Place of Birth		
South Korea	22	79
United States	6	21
Years in the US		
< 10	4	14
11–20	8	29
> 20	16	57

We evaluated how long the participants had been aware of their infection status. As shown in Table 2, an overwhelming majority had known that they were infected 10 to 20 years prior to this study. Approximately 70% of the participants had a history of CHB in their immediate family. Sixty-one percent were college graduates, and the rest were high school graduates. A little over half of the participants (57%) had health insurance.

**Table 2:** CHB History and Characteristics of HBsAg-seropositive participants

	Number of participants	Percentage (%)
CHB First Diagnosed		
Before 2000	21	75
2000–2010	4	14
After 2010	3	11
Family History^a^		
Yes	19	68
No	8	26
Do not know	1	4
Level of Education^b^		
High School	11	39
College	17	61
Health Insurance		
Yes	16	57
No	12	43
Currently Seeing Physician		
Yes	8	29
No	20	71

^a^refers to presence or absence of chronic hepatitis B in immediate family members (parents, children and siblings)

^b^Education levels are categorized as High school (those who graduated from high schools) and College (those who had college or post-graduate degrees)

Despite their long histories of infection, only 8 of 28 (29%) were currently seeing physicians for CHB care, and the remaining 20 (71%) were not seeing physicians or linked to any health care services (Table 2). All participants admitted that they had been recommended at least once to see a physician for a full evaluation and follow up when they were initially diagnosed. Of those 8 participants currently seeing physicians, all but one was diagnosed before 2010. Upon further evaluation, 6 of the 20 participants who were not currently linked to care had seen a physician at least once between their initial diagnosis and the time of the current investigation but failed to follow up due to various reasons. The most common reason was that they felt no symptoms and thus felt no need to seek care.

### Themes identified through content analysis

Low risk perception and knowledge of CHB is the first of three prominent themes we identified in the focus group data. The specific responses generated from these discussions underwent integrated analysis, and their main findings are summarized in Table 3.

**Table 3:** Lack of knowledge and risk perception for CHB and its complications

Theme	Question	Illustrative quotes
Part A. Asymptomatic nature of CHB	Do you know what symptoms CHB can give? If there are no symptoms, does this mean you are safe?	*Since my HBV infection diagnosis 15 years ago, I have never bothered to see a doctor for it. I never had any symptoms and I have never felt sick except for common colds. If I have symptoms such as easy fatigue or persistent tiredness, I would probably seek a doctor to have a checkup.*
Part B. Chronic nature of CHB	Have you heard of active vs. inactive disease? What do you know about them?	*Although I was diagnosed with chronic hepatitis B more than 20 years ago, I never bothered to see a doctor for a checkup. I remember my doctor then telling me that I was a healthy carrier, and I naturally thought I would not have any serious outcomes from this infection.* *I understand that because my HBV infection is inactive, I don’t need to worry about transmission. It made me feel good since I would not endanger anyone.* *I remember my CHB was once active when I was first diagnosed, but then in a follow-up, my doctor told me that my infection became inactive subsequently and there was no need to treat it. Since then, I felt no further need to see a doctor for my HB infection.*
	Is there a treatment for CHB?	*I know there are medicine for CHB, but I don’t think they can cure CHB. So, I don’t consider them as treatment.* *Heard there is no cure.* *Don’t think it’s curable for now- Believe new tx will come out soon.* *I haven’t seen any ads for treatment of Hep B-- believe there is no tx for hep B.*
Part C. Liver cancer and other complications	Do you know if chronic HBV infection can cause liver cancer and other complications?	*If I’m lucky then nothing will happen, but If I am unluckyn, then it will cause other complications* *I know that CHB can cause liver cancer, but I always felt that it was very rare. I know alcohol drinking can cause bad liver disease.* *I am not an alcohol drinker, so I am not really worried about liver cancer.* *Some will develop liver cancer or liver cirrhosis.* *Never thought about it. I would say higher chance of getting complications.* *I think liver cancer can happen only to those who are very sick with CHB, with a lot of symptoms and active infection.*

#### Low risk perception and knowledge of CHB

Participants had poor knowledge of CHB and showed a significant lack of understanding regarding the fact that the absence of symptoms can often be associated with a serious progression of the disease. For example, many participants falsely believed that if they showed no symptoms, it meant that their CHB was not active (Table 3 Part A). Most participants were also unaware that asymptomatic disease can revert to active disease (Table 3 Part B). Furthermore, the participants’ level of knowledge on antiviral treatments was low. Participants were either unaware of the existence of effective treatments or had little knowledge of the efficacy of existing treatments. When asked whether they knew if there was any medical treatment for HBV, an overwhelming majority of participants responded that there was no effective treatment (Table 3 Part B). While about half of the participants knew that antiviral medications for CHB existed, they did not consider them as treatment.

Focus group discussions also touched on the perceived risk for liver cancer, with only half of all participants recognizing the heightened risk for liver cancer among Asian Americans. While some participants were aware that CHB could lead to liver cancer, they were unaware that symptoms are uncommon in the early stages of liver cancer, and that liver cancer can be diagnosed in patients without symptoms (Table 3 Part C).

We additionally found that a majority of the participants were unaware of the need for preventive check-ups. They felt doctors’ office visits were only needed when they experienced physical symptoms of discomfort or pain. As one participant added, “*I was doing fine so I chose not to go back to the clinic*”; they felt the need to seek care only for urgent matters or illnesses but not otherwise (Table 4). Some patients were quicker to turn to Eastern medicine acupuncturists for herbal medicinal drinks to “*cleanse their body*.”

**Table 4:** Language, Immigrant status, and Stigma. *Impact of language and sociocultural factors on health decision making and health behaviors*

Theme	Question	Illustrative quotes
Language	How difficult is it to communicate with your providers who don’t speak Korean?	*I went to see a Chinese doctor, and we had to communicate in English. It was difficult for me to explain my symptoms clearly, and I was not certain if he understood me correctly.* *I have lived in the United States for the past 32 years, and I consider my English fairly good. However, I find it challenging to explain my concerns about CHB in English. Some of the things often expressed in Korean are expressed very differently from English. You cannot literally translate word for word and expect the meaning to be the same.* *I only looked for Korean doctors. I have never looked for American doctors due to the language barrier that I knew would be a problem.* *I do have a fear of interaction with non-Korean doctors at hospitals. I am not sure how to describe the symptoms I have, and I think because of this, I fear that there will be misunderstandings both ways.* *I went to see a doctor and there was an interpreter, who translated for me. Doctor did not ask many questions, and I am not sure if he understood me.*
Sociocultural	What are the personal issues that affect you when you plan to see a doctor?	*Inter-cultural misunderstanding:* *Doctor I met for hepatitis B explained that I need to come to clinic at least 1–2 times a year to make sure nothing bad has happened in my liver. But I am doing fine so I chose not to go back to the clinic.* *I do not see why I need a checkup unless I have a symptom.* *I visit my Eastern medicine acupuncturist for my regular well-being check ups, and I only visit doctor’s office when I am sick.* *Stress and financial with immigrant life:* *I definitely feel a certain type of burden when I go to the hospital. I feel that people at the hospital were not as nice as I thought they would be, and I wondered to myself, “Is it because I’m a minority that I’m getting a certain type of treatment?”* *I used to be a banker in Korea, but I cannot get a decent job here in the US. Fortunately, my wife is a nurse and has a good job. I feel depressed and thinking about going to see a doctor for my liver is not in my mind.* *Healthcare cost is too high in the US. My employer does not provide health insurance for every employee. And I can’t afford insurance now.* Structural: *Many of my Korean friends and I rely on the resources from Korean Community Center. I even get my sugar checked there. They provide me with the information about the doctors, pharmacies and hospitals.* *I was referred to a doctor at a university hospital, but it would take 2 months to see him, so I ended up not going.*
Stigma	What are the personal things you worry about because you are chronically infected?	*When I first got diagnosed, I became more careful around other people. I even ate at a different table because I didn’t want to affect my children.* *I have a girlfriend right now, and I am very troubled since she doesn’t know that I am HB infected. If I tell her, I don’t know how she would react. I’m afraid to tell the truth, so in a way, I feel shame...My doctor told me that if she is vaccinated, it wouldn’t affect her. I mean, there is no chance that she would get hepatitis B from me. But even then, I find it difficult to tell her the truth.*

#### Language, immigrant status, and stigma

The role of language barriers was evident through discussion. Regardless of the participants’ levels of education or length of residence in the U.S., language remained a barrier when it came to healthcare. While many participants could carry out normal, daily communication in English with relative ease, they recounted the difficulties in describing their medical symptoms and concerns. One participant, for instance, said, *“You cannot literally translate word for word and expect the meaning to be the same.”* Many ideas expressed commonly in Korean do not have English equivalents, potentially representing a language gap that can lead to miscommunication (Table 4).

The participants were asked whether they felt either disadvantaged or discriminated against when accessing healthcare as immigrants. Most participants responded similarly, saying that they felt some degree of separation or alienation from healthcare professionals especially during their experiences in hospital settings. Some participants recalled a sense of burden that came along with having to interact with people who did not speak Korean, and the resulting fear of misunderstanding and lack of trust that affected their decisions to forego seeking healthcare again (Table 4).

In addition, the stress associated with immigrant life further complicated the processes required for adequate access to care (Table 4). This stress stemmed from personal internalization of minority ethnic group membership and manifested as uncertainty and skepticism towards the healthcare system, as noted by a participant who wondered if they were receiving differential treatment based on their membership in a minority group.

Stigma-associated events were evident and serious according to some participants. Most participants were familiar with the stigma attached to CHB. Several participants elaborated that as a result of their diagnosis, they became afraid to socialize openly, go out on dates, or even interact with their family. One participant often felt guilty eating with his children at the same table. Although this participant demonstrated a lack of knowledge on the routes of HBV transmission (i.e., the fact that HBV is not transmitted through sharing food), his behavior around his own children demonstrates the reality of stigma. Another participant who was younger, in his thirties, explained his agony over telling his girlfriend about his CHB status, as he thought it would cause strain and anxiety in his relationship.

#### Financial and institutional barriers

Financial issues were cited as common reasons for delayed visits to doctor’s offices. Participants cited a lack of insurance as the main reason they were not currently seeing a doctor. They also described difficulties in enrolling and accessing insurance and particularly, issues with the cost of enrollment (Table 5). One participant said, “*The insurance cost keeps rising, and the high copay and deductible payments even with insurance is way too much to afford. So I do not have any health insurance.”*

**Table 5:** Financial and related healthcare system barriers

Theme	Question	Illustrative quotes
Financial	Do you have health insurance that covers doctor’s visit?	*I was able to see a doctor regularly until about 10 years ago when I still worked in a large corporation. I had health insurance then. But now, with the insurance cost rising so high, I am not able to afford it. I am 62 years old now, and I will get my Medicare in three years.* *I had Obamacare until a year ago. The insurance cost keeps rising, and the high copay and deductible payments even with insurance is way too much to afford. So I do not have any health insurance. Fortunately, I don’t feel sick, and I hope my HBV doesn’t cause any serious problem. I know I need to have a checkup soon, though* *Financial problems are definitely the greatest issue when it comes to seeing a doctor or going to a hospital*
Healthcare system barriers	Have you had any issues with doctors or hospitals you experienced in the United States?	*I have been to two doctors in the past several years in the United States. I was not impressed with their level of knowledge and care on CHB. I am not sure how familiar the doctors in the United States are with HBV disease.* *I think hospital and staff in the United States are not familiar with CHB. I also heard that the national health policy to screen HBV in Asians was only recently instituted.* *Hospitals in Korea have different systems [than those in the US] that make it easier to seek out care. For example, Korea hardly takes appointments and even when they do, you don’t have to wait long.* *I went to see an American doctor, who was not familiar with the treatment options for CHB. He referred me to another doctor at a university hospital, but it would take two months to see him, so I ended up not going.* *As a non-native, it’s difficult to find doctors. Korean community centers and media are the only means for me to find doctors.*

One participant who had previously worked as a nurse in China, a country where hepatitis B and liver disease are highly prevalent, felt that doctors in the U.S. were less knowledgeable about CHB and as a result did not empathize well with patients, often appearing almost negligent of patients and their needs. Similarly, one participant stated that she did not feel comfortable or cared for when she visited her doctor’s office. Another issue raised was the lack of information that doctors and health professionals would relay to CHB patients (Table 5). Due to this perceived lack of medical expertise in the community, participants felt discouraged from pursuing follow-up care.

In addition, a number of participants remarked on how “complicated” hospitals seemed to be in the U.S., involving lengthy processes such as verifying identification in multiple forms and going back and forth between different wings of the hospital, causing “unnecessary” delays and eventually discouraging future visits. They noted, for instance, that at a majority of hospitals and doctor’s offices in South Korea, appointments are not necessary; access to care is first come, first served. In contrast, appointments at doctor’s offices or hospitals are often not available for several weeks or even months in the U.S.

## Discussion

Our findings reveal a significant lack of access to CHB care in a group of 28 Korean American participants with CHB. The results have identified an important set of barriers to health literacy, which are largely informed by the sociocultural overlay of the participants’ experiences. Low risk perception and knowledge, cultural differences and stigma, and financial and institutional barriers, which were identified as prominent issues in focus group discussions, all affected health literacy and influenced the participants’ capacity to access care.

### Health literacy

The causes of low health literacy levels pertaining to CHB among high risk populations in the U.S. are multifactorial and include linguistic and culture-specific factors, both of which influence participants’ access to and experience with healthcare. For instance, our results showed that although a majority (86%) of the participants had been living in the U.S. for over 10 years (Table 1), there was a consensus that linguistic and cultural differences significantly hindered their healthcare-seeking abilities (Table 4). To complicate matters more, language used among healthcare professionals often containing medical jargon further compromised participants’ health literacy, resulting in poorer healthcare experiences. The participants’ ethnic and cultural identity clearly continued to shape their behaviors, attitudes and perceptions, which was evident through their low utilization of healthcare.

The concept of distributed health literacy, which refers to how health literacy skills and practices are distributed through social networks [36, 37], is applicable to this study’s context. The participants in this study may very well draw on their social networks for support with certain health-literacy related tasks such as interacting with health professionals and making decisions about their health. The distribution of health literacy has been shown to positively impact health management and related decision-making processes [38]. Given the participants’ infrequent use of healthcare services revealed by this study, distributed literacy may not be in strong effect in this specific population. Future studies may look further into the role of specific social networks in facilitating health related experiences. This may point to a greater need for community-based programs that focus on using existing social structures to maximize impact in behavioral and health change across communities.

Another potential barrier to health literacy among the participants was their lack of familiarity with the concept of preventive medicine. Being unfamiliar with medical concepts is a characteristic of low health literacy and can contribute to the communication gap between patients and providers [39]. Patients with low health literacy as a result of poor health knowledge are also more likely to ask fewer questions, which widens the knowledge gap and is associated with adverse health outcomes [40].

An individual’s social and cultural contexts are inextricably linked to how they perceive and act on health information. The results of this study show how culture and language help set the stage for application of health literacy skills. The participants often linked their actions to the presence of cultural differences that deterred them from following through on health-related tasks. Cultural mismatches have been shown to contribute to disparities in health experienced by racial, ethnic, and minority groups [41, 42]. Additional studies have shown that when controlling for health literacy, racial and ethnic disparities in health care quality and outcomes disappear [43]. Low health literacy is in turn a key cause and effect of these disparities.

Stigma also played an important role in generating negative health behaviors in CHB. The manifestation of stigma through cultural reinforcement resulted in establishing shame, disgrace, and humiliation among those with CHB. This led to participants deliberately choosing not to seek care, leading to delayed monitoring of CHB conditions, treatment, and critical disease maintenance (Table 4). In addition, a lack of health literacy could have potentially contributed to experiences with stigma, resulting in negative health consequences.

### Healthcare systems and providers

Healthcare providers and public health systems play critical roles in governing health literacy since they can affect the degree and ease with which people find and utilize healthcare systems and services. A lack of knowledge among healthcare providers across various aspects of CHB such as the prevalence of HBV in high-risk populations, clinical sequelae, and management can be detrimental for populations at risk. While detailed guidelines on these topics are already in place and updated regularly, studies continue to point to a lack of knowledge among health care providers as well as a failure to follow these guidelines [44]. A survey of primary care providers in San Francisco [45] showed that 30% of respondents were not able to identify the correct test to use for diagnosing chronic HBV infection. In addition, Chu [46] found that only 18–30% of Asian American primary care providers who treat Asian American adult patients reported routinely testing them for HBV infection in their practices.

In agreement with these studies, a majority of the participants in the present study said they were not satisfied with the care they had received. Many also felt that there was a lack of medical expertise in their communities. Participants were often unsure of where to access care but mentioned that community centers were helpful in locating doctors within the community (Table 5).

### Culturally competent interventions for CHB

The Institute of Medicine [47] has recognized that health disparities result from a lack of cultural competence in care and gaps in health literacy and has recommended strategic approaches to training that will help prepare health professionals to recognize and take on these problems. This study highlighted numerous culture-specific barriers to healthcare, emphasizing the need for culturally competent health services, especially in today’s increasingly diverse population. These barriers elucidated the lack of health literacy and negative health behaviors among the study population.

To counter the effects of barriers to health literacy and access to care, innovative health and educational services should be designed to account for the intricacies in health literacy approaches targeting diverse populations [48, 49]. In fact, various novel initiatives and strategies have been employed to resolve health literacy deficits with respect to CHB. An intervention created YouTube channels aimed at facilitating the understanding of the history and pathophysiology of CHB [50]. In addition, culturally appropriate photonovels have been developed to prevent CHB and liver cancer [51]. These photonovels feature culturally tailored components, such as faces of people in the same ethnic group playing central characters in the stories, and storylines drawn from common cultural experiences. This approach has been effectively used as a means of participatory education that maximizes the learning experience through active involvement of the target population.

Community-based initiatives can also play a crucial role in mitigating deficiencies in health literacy [52, 53]. As pointed out by participants, KCS, which provided the HBV screening and educational programs served as a major resource to obtain healthcare needs (Tables 4 and 5). KCS, for instance, offered extensive services including screening programs, vaccination, and linkage to Korean-speaking physicians in their community who could provide culturally competent care.

### Limitations of the study

There are several limitations to this study. The current study population age ranged between 20 and 69, excluding younger and older subjects. Thus, the under- or over-representation of certain age groups in this study could have affected the results, as not all age groups face the same barriers, and perceptions of healthcare interactions also vary by age [54]**.** Another limitation is that this study could not account for the Korean American CHB patients who do not visit KCS and similar programs. These harder to reach populations may have different barriers to accessing care that were not detected in this study.

## Conclusion

Asian Americans are highly vulnerable to poor health outcomes from chronic HBV. We have identified and evaluated self-perceived sociocultural barriers to CHB care in an Asian American population. These barriers impact the community’s health literacy, influencing the levels of healthcare utilization and of screening and preventative behaviors. These findings may help inform future strategies and programs built around culturally competent health literacy frameworks. Given the significant lack of access to care in the culturally tailored program these participants were attending, there may be factors both related and unrelated to health literacy that hinder the proper use of these resources and the advancement of care. This warrants further exploration of the multidimensional nature of health literacy, an essential skillset which allows people to make decisions about their health and serves as the gateway to optimal health.

## Supplementary Information

**Additional file 1.** HBV Demographic Form. Survey assessing demographic and epidemiologic characteristics of participants.

## Data Availability

The datasets used and/or analyzed during the current study are available from the corresponding author on reasonable request.

## Abbreviations

CHB: Chronic hepatitis B
HbsAg: Hepatitis B surface antigen seropositive
HBV: Hepatitis B virus
KCS: Korean Community Services
