# Supplementary material for: Sociocultural barriers to hepatitis B health literacy in an immigrant population: a focus group study in Korean Americans
Source: BMC Public Health. 2021 Feb 25;21:404. doi: 10.1186/s12889-021-10441-4 (PMC7908637; doi:10.1186/s12889-021-10441-4)
Supplement: Supplementary file 1 — Additional file 1. HBV Demographic Form. Survey assessing demographic and epidemiologic characteristics of participants. [file 12889_2021_10441_MOESM1_ESM.docx]

**HBV Focus Group Study Demographic Form**

Age: ____________________________

Birthplace: _______________________

Gender:

- - Female
  - Male
  - Prefer not to say

Years living in the US: __________________________

Preferred language of communication: _______________

Do you have health insurance?

- - Yes
  - No

When were you diagnosed chronic hepatitis B? __________________________________

Have you ever been evaluated by specialist regarding hepatitis B?

- - Yes
  - No

If yes, how long after the diagnosis did you seek consultation? _____________________

If not, what were the reasons? ____________________________________________

Are you currently seeing a doctor for hepatitis B?

- - Yes
  - No

Are you currently taking any medication for hepatitis B?

- - Yes
  - No

Have you ever received any medication for hepatitis B?

- - Yes
  - No

Do you have family history of viral hepatitis B or any liver disease?

- - Yes
  - No

Do you have any other chronic illness (e.g., diabetes mellitus, hypertension, kidney disease, heart disease, etc.)?

- - Yes (please specify: ­­­­_______________________________________)
  - No
